# Seasonal Patterns of Resource Use Within Natural Populations of Burying Beetles

**DOI:** 10.1002/ece3.70429

**Published:** 2024-10-25

**Authors:** Swastika Issar, Chloé Leroy, Patrizia d'Ettorre, Rebecca M. Kilner

**Affiliations:** ^1^ Department of Zoology University of Cambridge Cambridge UK; ^2^ National Centre for Biological Sciences‐Tata Institute of Fundamental Research Bangalore India; ^3^ Laboratory of Experimental and Comparative Ethology (LEEC), UR4443 University Sorbonne Paris Nord Villetaneuse France

**Keywords:** age structure, burying beetles, cuticular hydrocarbons, phenology, reproductive success, seasonality

## Abstract

For organisms in temperate environments, seasonal variation in resource availability and weather conditions exert fluctuating selection pressures on survival and fitness, resulting in diverse adaptive responses. By manipulating resource availability on a local spatial scale, we studied seasonal patterns of resource use within natural populations of burying beetles *Nicrophorus vespilloides* in a Norfolk woodland. Burying beetles are necrophagous insects that breed on vertebrate carcasses. They are active in Europe between April and October, after which they burrow into the soil and overwinter. Using breeding and chemical analyses, we compared the fecundity and physiological state of beetles that differed in their seasonal resource use. We found seasonal variation in carrion use by wild burying beetles and correlated differences in their reproductive success and cuticular hydrocarbon profiles. Our results provide novel insight into the seasonal correlates of behaviour, physiology and life history in burying beetles.

## Graphical abstract

We present experimental evidence of seasonal variation in resource use within natural populations of a necrophagous insect that requires small vertebrate carrion for reproduction. Seasonal patterns of carrion use by wild burying beetles are associated with differences in their reproductive success and cuticular hydrocarbon profiles. Our results provide novel insight into the seasonal correlates of behaviour, physiology and life history in burying beetles.

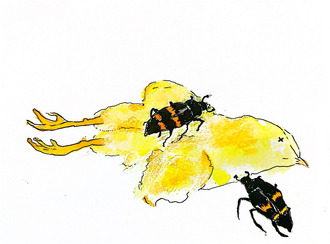

## 1 Introduction

Seasonality can be a strong and critical source of environmental variability for organisms in temperate environments (Williams et al. 2017). It can impose fluctuating selection pressures on survival and fecundity that give rise to a great diversity of adaptive responses (Varpe 2017; Zhang et al. 2019) leading to temporal variations in fitness (e.g. Ohgushi 1991) that contribute to population dynamics (Morgan, Walters, and Aegerter 2001; Ragland and Kingsolver 2008; Johnson et al. 2016). However, seasonal cycles are steadily being disrupted by climate change, resulting in climate‐induced extinctions, distributional and phenological changes and species' range shifts in wild populations (Easterling et al. 2000). Theoretical predictions suggest that phenological shifts could be highly variable both among and within populations because the consequences of climatic change could vary across the geographical range of a species and impact individuals differentially (Heard, Riskin, and Flight 2012).

Understanding the ecology and evolution of phenological traits in interacting species is therefore key to developing a deeper understanding of how climate change affects species persistence and biodiversity. This is particularly true when seasonality in reproduction critically depends on the availability of key resources, which themselves vary in abundance during the year. In some cases, we know that populations can potentially respond to climate change because they have already shown themselves to be capable of adaptive phenological change. An example comes from the apple maggot fly *Rhagoletis pomonella* (Bush 1969; Mattsson et al. 2015). In this species, the timing of fly reproduction is critically dependent on host fruits which become the mating arena for adults and then the diet for their developing larvae. In the last two centuries, *R. pomenella* has evolved into a new race that specialises in breeding on apples rather than the ancestral hawthorn host fruit. It has achieved this host shift through a phenological shift in its reproduction to coincide with the earlier fruiting time of apples (Egan et al. 2015). In other cases, though, adaptation like this might not be possible, or feasible in the short‐term. Intraspecific variation in phenological responses to climate change could then pose a threat to ecosystem functioning by potentially desynchronising and disrupting ecological interactions (Heard, Riskin, and Flight 2012; Thackeray et al. 2016) and ecosystem functioning (Grimm et al. 2013; Schmitz 2013). The natural history of most species is insufficiently well‐known to predict which of these outcomes is more likely.

Here we describe seasonal patterns of reproduction in the burying beetle, *Nicrophorus vespilloides*. Burying beetles (*Nicrophorus* species) are necrophagous insects that live in seasonal environments. Burying beetles are completely absent from sub‐Saharan Africa, Australia and Antarctica and, with the exception of a few tropical areas where this lineage occurs, they tend to be found in cool habitats at higher elevations (Sikes 2005, Sikes and Venables 2013, Merritt & De Jong 2015). They breed in Europe and North America between April and October, with variation in abundance depending on the species (Dekeirsschieter et al. 2011). At the end of their breeding period, burying beetles burrow into the soil and overwinter as adults or in the pre‐pupal stage for some species (Pukowski 1933; Peck and Kaulbars 1987; Ratcliffe 1996). Burying beetles breed on small vertebrate carcasses and therefore serve a key ecological function in decomposition and nutrient recycling. Their carrion breeding resource acts both as a mating arena for adult beetles and a food resource for developing larvae but it is ephemeral and unpredictably distributed (Scott 1998). Carrion can be scarce, making competition among burying beetles to secure ownership correspondingly intense. Through an elaborate system of biparental care (Milne and Milne 1976), burying beetles conceal carrion from rivals and defend it from attack. Together the pair removes any fur or feathers, rolls the flesh into a ball, covers it in antimicrobial fluids and buries it below ground where it becomes an edible nest for the developing larvae.

Seasonality in reproductive behaviour varies greatly among *Nicrophorus* species. While *N. vespillo* populations studied by Meierhofer, Horst, and Müller (1999) in Bielefeld, Germany, did not differ in the number of offspring produced throughout the season, the period of parental care was significantly higher in spring, compared to early or late summer. This is likely mediated by lower temperatures in spring, which slow down offspring development and therefore prolong the period during which offspring require parental defence from attack. Furthermore, natural populations of *N. orbicollis* in southern New Hampshire, United States produced heavier broods in the first few weeks of the breeding season compared to later broods (Scott & Traniello 1990). This could be attributed to less intense competition with flies at the beginning of the season or, potentially, to strategically greater levels of investment in first broods. In another work, Wilson and Fudge (1984) sampled two different sites in Michigan, United States using large and small mice carcasses, and found a large amount of unexplained variation in brood size. At one of the sampling sites, *N. orbicollis* beetles had fewer offspring in early summer (June) while *N. defodiens* had fewer offspring in late summer (August).

We investigated whether seasonal variation in reproductive behaviour was linked to seasonal variation in resource use within a wild population of *N. vespilloides*. Recent work (Wettlaufer et al. 2021) on carrion beetles (Silphidae) in south‐eastern Ontario, Canada suggests that competition for carrion may have led to resource partitioning between ecologically similar species through seasonal differences in beetle activity and abundance. Across a broad geographical area, burying beetle species and populations also appear to have differentially adapted to breed on different species of vertebrates, depending on local vertebrate diversity (Wilson and Fudge 1984; Hocking et al. 2007). There is one report of differential resource use involving sympatric species of burying beetles – *N. investigator* and *N. defodiens* – specialising in aquatic versus terrestrial carrion (Hocking et al. 2007). Together these studies suggest that resource use influences niche partitioning among species, though it is unknown whether similar patterns exist within species.

This led us to hypothesise that similar seasonal resource‐based partitioning could potentially happen within *N. vespilloides*, since the relative abundance of mammalian and avian carcasses available to the beetles potentially varies across the beetle breeding season. In the UK, there is considerable mortality among fledgling songbirds in late spring and early summer (Newton 1998; Chase, Nur, and Geupel 2005; Clapham 2011; Capstick 2017) whereas rodent populations show high mortality in mid‐late summer (Moffat 1910; Harris 1979; Merritt, Lima, and Bozinovic 2001; Haberl and Kryštufek 2003; Clapham 2011). The adaptive partitioning of resources within a population, driven by seasonal variation in resource availability, could lead to differences in reproductive success if sub‐populations become temporally separated and specialise on different carrion types.

We further hypothesised that seasonal variations in resource use could be manifested in a beetle's cuticular hydrocarbons, which could then potentially be used as a marker for identifying any resource specialisation that exists between subpopulations (Haberl and Kryštufek 2003; Chase, Nur, and Geupel 2005). Cuticular hydrocarbons (CHCs) are hydrophobic compounds present in the arthropod cuticle. They have been shown to evolve locally and adaptively to environmental variation in geography, latitude and seasonality experienced by natural populations of *D. melanogaster* (Ingleby 2015; Rajpurohit, Zhao, and Schmidt 2017). Furthermore, Steiger et al. (2007) found that beetles maintained on a diet of insects versus vertebrate carrion differed significantly in their cuticular signatures. Cuticular hydrocarbons also have been known to differ based on dietary resources in several insect species and can facilitate differential mating (Liang and Silverman 2000; Buczkowski et al. 2005; Ferveur 2005; Chung and Carroll 2015). Since the carrion resources available to burying beetles for feeding and reproduction vary considerably across the entire field season, it is possible that these seasonal variations in resource use are also manifested in their cuticular hydrocarbons (Haberl and Kryštufek 2003; Chase, Nur, and Geupel 2005; Clapham 2011).

We tested these hypotheses by investigating seasonal patterns of resource use within a natural population of *N. vespilloides* in Thetford Forest, Norfolk, UK. We first tested for evidence of seasonality in resource use by investigating whether *N. vespilloides* beetles from early summer (June) were more likely to be trapped on mice or chick carrion compared with those from late summer (August). Next, with laboratory experiments, we tested which carrion type yielded the greater reproductive success and whether this differed between beetles that were trapped in June versus August. Finally, we tested whether beetles that were attracted to different types of carrion during the early, mid, and late field seasons also differed predictably and seasonally in their cuticular hydrocarbons (CHCs).

## 2 Materials and Methods

### 2.1 Is There Seasonal Variation in the Trapping Frequency of Burying Beetles on Chick and Mice Carrion Between June and August?

#### 2.1.1 Study Area and Trapping Methods

**FIGURE A1:**
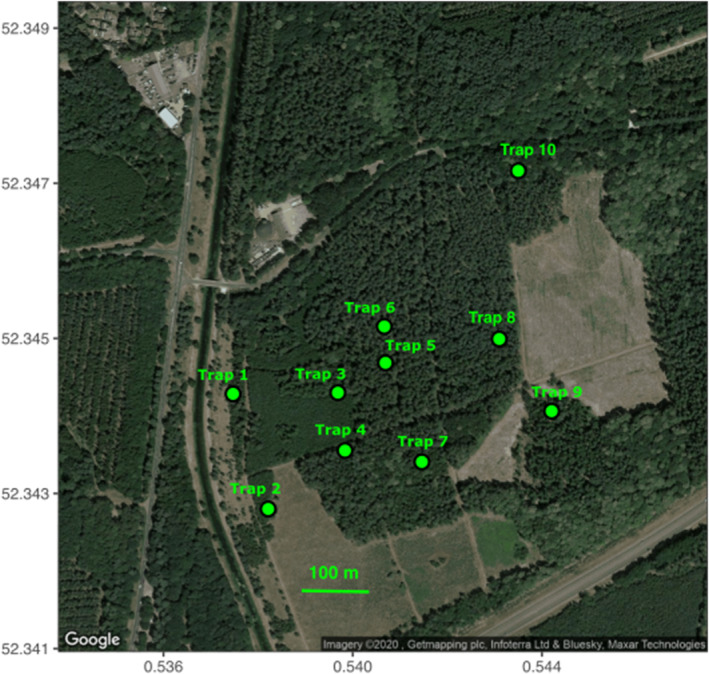
Beetle trapping locations in Thetford Forest. The coordinates of each location are represented using the longitude value on the horizontal axis and the latitude value on the vertical axis.

We sampled the burying beetle population at Thetford Forest (52°20′39.5″ N 0°32′14.9″ W), Norfolk, UK from May to October 2017 at the trap locations shown in Figure A1, under permit from Forestry Commission England. We used carrion‐baited beetle traps (Japanese Beetle Trap Kit from Scotts Co., not treated with any pheromones), suspended in vegetation 1–2 m above ground. The bottom half of the trap was filled with Miracle‐Gro compost, and a small dead vertebrate was placed on the top as bait. The contents of the trap were collected at intervals and brought back to the lab for processing. The trap was then refilled and rebaited. After processing in the lab, no beetles were released back into the field.

Beetles were sampled using a paired‐trap arrangement, in which we placed two beetle traps – one baited with a dead domestic chick and the other baited with a dead mouse – near each other at each trap location and recorded the number of beetles found in each trap. The traps within each experimental pair were placed 1–2 m apart. Beetles locate carrion using olfactory cues when in flight (Potticary et al. 2024). The traps are placed above ground to ensure optimal diffusion of cues from the carrion, which can remain undisturbed by other animals on the ground. Pairs of traps were placed 200–400 m apart from each other. With this design, beetles were given a simultaneous choice between a dead mouse and a dead chick. Each time we rebaited a trap with carrion (every 10–15 days throughout the field season), we rebaited it with the alternate carrion type. Therefore, if a mouse carcass had been placed in the trap previously, it was replaced by a chick carcass on the next sampling trip to ensure that the trap location itself did not bias beetle catch. The mice and chick carcasses used were matched in weight (30–40 g).

### 2.2 Processing Field‐Caught Beetles

At the lab, we used carbon dioxide to immobilise each beetle and brush off any mites stuck to it. We recorded the pronotum width and sex of each *N. vespilloides* beetle we trapped. We compared beetles collected at two different time points during the burying beetle season: the first set was collected in June 2017 after 10 days of trapping between 4 June and 14 June, and the second set was collected in August 2017 after 15 days of trapping between 4 August and 19 August. The 10 trapping locations (Figure A1) were the same across both sampling periods.

### 2.3 Does Reproductive Success Vary With Carrion Substrate and/or Season?

#### 2.3.1 Measuring Reproductive Performance

After collecting beetles from the traps, measuring and identifying them, we put each *N. vespilloides* individual into its own personal small plastic box (12 cm × 8 cm × 2 cm) and fed it 1 g of beef mince. The beetles were stored alone in their boxes for 7–10 days to ensure that any newly eclosed individuals had had sufficient time to become sexually mature before we measured their reproductive performance.

For breeding, we placed a pair of beetles (one male and one female) in a larger plastic breeding (17 cm × 12 cm × 6 cm) box half‐filled with Miracle‐Gro compost and provided with either a chick or mouse carcass that had been freshly thawed out and had not yet begun decomposing. Each member of the pair had been trapped on the same type of carrion and we bred them on the same carrion they were trapped upon. This method was used twice, once for beetles collected in June and once for those collected in August, yielding four treatments in all.

The mass of the carcass provided for reproduction was recorded and kept consistent within each treatment. We then placed the breeding box inside a cupboard so that it was shielded from light to mimic the low light conditions typically experienced by beetles as they breed below ground. The cupboards were maintained in an air‐conditioned temperature‐ and humidity‐controlled lab environment that was monitored constantly throughout the year. Eight days after pairing the beetles (by which point the larvae had completed development and were starting to disperse away from the remains of the carcass), we counted and weighed the surviving larvae from each pair.

We used the following measures to record reproductive success in our experiments: (1) Brood failure: We recorded the total number of broods that failed to produce any larvae. ‘0’ denoted broods that failed and ‘1’ denoted those that had at least one surviving larva at 8 days post dispersal. (2) Brood size: The total number of dispersing larvae 8 days post breeding. (3) Average larval mass: Total mass of the brood at dispersal (g) divided by the brood size. (4) Larval density: Brood size divided by the mass of the carrion used for breeding (g). (5) Carcass use efficiency:

$$\left[\text{Total brood mass}\;\left(g\right)\;\text{divided}\;\mathrm{by}\;\text{original carrion mass}\;\left(g\right)\right]\times 100\%$$

In June, 53 pairs of beetles trapped on mice (MM) and 24 pairs of beetles trapped on chicks (CC) successfully produced broods with at least one larva. There were 4 failed broods (3 on mice carcasses and 1 on a chick carcass). In August, 16 pairs of beetles trapped on mice (MM) and 25 pairs of beetles trapped on chicks (CC) produced broods with at least one larva. There were 7 failed broods (2 on mice carcasses and 5 on chick carcasses). The failed broods were excluded from analyses involving any measures of reproductive success other than brood failure.

### 2.4 Do Beetles That Are Attracted to Different Types of Carrion Also Differ Predictably and Seasonally in Their CHCs?

For this experiment, we sampled a total of 63 females; 32 were trapped on chicks and 31 were trapped on mice. Forty females were collected on 23 May 2017 (‘early’ season: 20 on chicks and 20 on mice). Six females were collected on 14 June 2017 (‘mid’ season: 3 on chicks and 3 on mice). Seventeen females were collected on 4 September 2017 (‘late’ season: 9 on chicks and 8 on mice). After removing the mites from the body of the beetles, we isolated up to two female beetles from each trap individually in a glass vial for 15–20 min before storing them in a fresh vial at −80°C. Later, we processed the beetles for CHC extraction by allowing them to thaw at room temperature for 30 min. We then soaked them in 4 mL of solvent (99% hexane, HPLC grade) for 20 min. We transferred the extract obtained to a clean vial and allowed it to evaporate completely in a fume hood under nitrogen gas. At this stage, the sealed vials were shipped to Prof. Patrizia d'Ettorre's lab at Université Sorbonne Paris Nord for analysis and characterisation.

#### 2.4.1 CHC Analysis and Characterisation

We resuspended the extract in 400 μL of pentane (HPLC grade) and added an internal standard (C18, Octadecane at 16 ng/μL) to each extract. The internal standard was used to determine the absolute amount of cuticular compounds present in each sample. We then analysed 2 μL of the extracts using GC–MS (Agilent Technologies 7890A gas‐chromatograph coupled to a 5975C Mass Spectrometer equipped with a HP5MS GC column (30 m × 0.25 mm × 0.25 μm) and operated at 70 eV in the electron impact ionisation mode). The carrier gas used was helium at 1 mL/min. The column oven was programmed as follows: an initial hold of 1 min at 70°C, then increased to 200°C at 35°C/min, to 320°C at 4°C/min (held for 20 min).

We identified cuticular hydrocarbons based on their retention times (compared to standards) and fragmentation patterns. We manually integrated the chromatograms and converted the peak areas of the total hydrocarbon fraction using the MSD ChemStation software by Agilent Technologies, Inc.

#### 2.4.2 Data Visualisation and Statistical Analysis

##### 2.4.2.1 Field and Reproductive Success Data

We carried out all statistical analyses to test our predictions using R (RStudio version 1.3.959) with generalised linear models (GLM) and generalised linear mixed models (GLMM) using the lme4, glmmsr and MASS packages. Analysis‐of‐variance tables for model objects were calculated using the ‘car’ package. Post hoc comparisons using Tukey's HSD test were carried out using the package ‘lsmeans’. The asymptotic test for the equality of coefficients of variation (CV) was carried out using the ‘cvequality’ package (Feltz and Miller 1996).

### 2.5 Is There Seasonal Variation in the Trapping Frequency of Burying Beetles on Chick and Mice Carrion Between June and August?

We calculated the average number of beetles per day by dividing the total number of *N. vespilloides* beetles found in a trap by the number of days the traps had been left out. We focussed on the two different time points for which we also measured reproductive outcome, namely June and August 2017, using a GLMM that included carrion type and sampling month as fixed effects, and trap ID and sampling date (to account for any differences in sampling effort) as random factors with a Poisson error structure. The total number of *N. vespilloides* beetles found in a trap on the sampling day was used as the response variable.

### 2.6 Does Reproductive Success Vary With Carrion Substrate and/or Season?

We examined the effect of month‐trapped, carcass type used for breeding and their interaction on the following measures of reproductive success: (1) brood success versus failure, using a multivariate logistic regression model with a binomial error term; (2) the number of dispersing larvae, using a GLM with a Poisson error term; (3) average larval mass using a linear model; (4) larval density using a linear model and (5) carcass use efficiency using a linear model.

When arriving at a minimal model using GLMs and GLMMs to explain our results, we removed non‐significant terms and interactions using stepwise elimination. When presenting the results from post hoc analyses, we list all the terms that were tested, and their statistics at the last point when they were retained in the model.

### 2.7 Do Beetles That Are Attracted to Different Types of Carrion Also Differ Predictably and Seasonally in Their CHCs?

**FIGURE A2:**
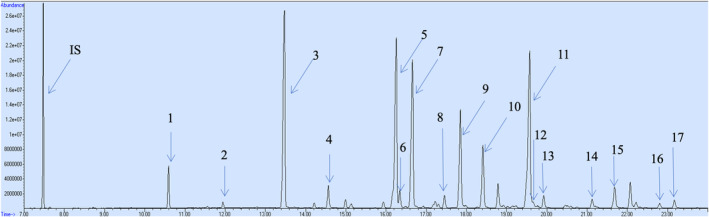
Gas‐chromatogram of the CHC profile of *N. vespilloides*, showing the 17 most regularly occurring peaks in our samples. The *x*‐axis represents retention time, and the *y*‐axis represents signal intensity. Identification of the peaks is provided in Table A1.

To analyse the chemical profile of both sets of beetles, we selected 17 most regularly occurring GC–MS peaks (Figure A2, Table A1). These represented the hydrocarbons we had identified and integrated using the MSD ChemStation software.

CHCs of field‐caught burying beetles could vary due to season of trapping, carcass type, individual quality, age, reproductive status and several abiotic factors (Howard and Blomquist 2005; Blomquist and Bagnères 2010). Therefore, we have used a hypothesis‐free clustering methodology that does not assume a priori a likely cause for variation in the CHC profiles of the beetles.

We carried out the principal component analysis, hierarchical clustering and visualisation of the data using *ggplot2*, *dplyr, pvclust, FactoMineR* and *factoextra* packages in R (RStudio version 1.3.959).

We log‐normalised the peak areas within each sample using the following formula (Aitchison 1982):

$$Z_{\mathrm{ij}}=\ln\left[\frac{Y_{\mathrm{ij}}}{g\left(Y_{j}\right)}\right]$$

where Z_*ij*_ is the transformed area of peak *i* for beetle *j*; *Y*_*ij*_ is the area of peak *i* for beetle *j* and g(*Y*_*j*_) is the geometric mean of the areas of all peaks for beetle *j*.

We used the standardised area values of the 17 peaks for hierarchical cluster analysis with Ward's classification method to classify our samples. The significance of each node in the cluster was determined by multiscale bootstrap clustering with 10,000 iterations using the ‘pvclust’ package in R (Suzuki and Shimodaira 2006). We set the confidence level for the *p*‐value threshold to 95% and ensured that only the most significant clusters (with a *p*‐value lower than 0.05) were highlighted (Figure 3).

To examine how the CHC compounds found in our samples contribute to the discrimination of these samples, we performed a principal component analysis (PCA) on the log‐normalised peak areas. We implemented the PCA using singular value decomposition (Hartmann, Krois, and Rudolph 2023) for better numerical accuracy. Plotting standard deviations of principal components (PCs) and proportion of variances against all 17 PCs, we used the elbow method to determine the optimal number of PCs that explained the maximum amount of variance in our data (Jolliffe 2002). Based on the visual inspection of the elbow plots, we retained the first 10 principal components, which capture most of the variance in the data (96.3%). We then visualised the data using a heatmap depicting the loadings of the first 10 PCs (Figure A4A). To check the correlation between the first two principal components and the original variables, we calculated the squared cosine value (cos2) for each variable by squaring the cosine of the angle between the vector with the variable's coordinates and the origin of the graph (Figure A4B).

Cluster validation of our data indicated one outlier (Sample M13E). We confirmed this visually by using a 2‐dimensional scatterplot before removing the outlier. We then repeated our PCA and clustering analysis without this data point.

## 3 Results

### 3.1 Is There Seasonal Variation in the Trapping Frequency of Burying Beetles on Chick and Mice Carrion Between June and August?

**FIGURE 1:**
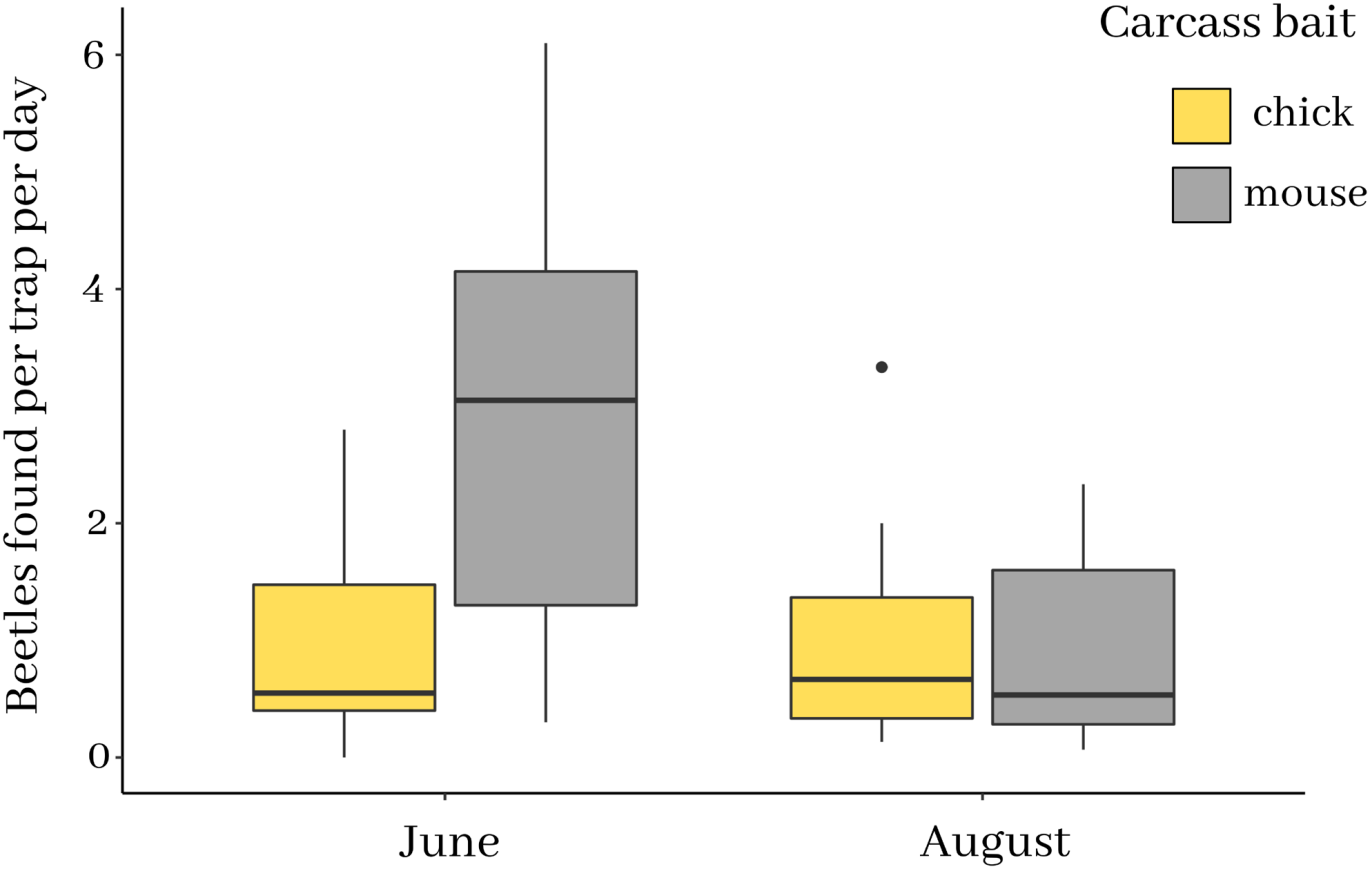
The number of *Nicrophorus vespilloides* beetles trapped on chick and mouse carrion at two time points during the 2017 field season. The number of beetles caught per trap per day in traps that were chick‐baited (yellow bars) and mouse‐baited (grey bars), from June 2017 (*N* = 391 beetles over 10 days) and August 2017 (*N* = 287 beetles over 15 days;). The box bounds represent the inter‐quartile range (IQR), the whiskers represent 1.5 × IQR, the central horizontal line is the median, and the single points are outliers in the data.

In June 2017, the mean catch per trap per day was 1 ± 0.29 (SEM – standard error of the mean) beetles on chick carcasses and 2.91 ± 0.60 (SEM) beetles on mice. In August 2017, the mean catch per day was 1.01 ± 0.32 (SEM) beetles on chick carcasses and 0.9 ± 0.27 (SEM) beetles on mice. There was a significant interaction between month and trap‐bait on the number of beetles caught (Figure 1, Table 1). In June, beetles were more likely to be caught on mice than on chicks (Tukey post hoc comparison: *z* ratio = −9.244, *p* < 0.0001), whereas by August they were similarly likely to be found on both sorts of carrion (Tukey post hoc comparison: *z* ratio = 1.006, *p* = 0.3144).

**TABLE 1:** Model summary showing results of GLMM to test for the effects of carrion type, sampling month and their interactions on the number of *Nicrophorus vespilloides* beetles trapped using avian versus mammalian carcasses in June and August 2017.

Fixed effects	Estimate	SE	*z* value	*p*r(> |*z*|)
Intercept	2.6213	0.1729	15.163	< 2e‐16***
Carcass‐Mouse	−0.1186	0.1179	−1.006	0.31436
Month‐June	−0.4187	0.1284	−3.262	0.00111**
Carcass‐Mouse × Month‐June	1.0681	0.1155	9.244	< 2e‐16***

Significance codes: 0 ‘***’ 0.001 ‘**’ 0.01 ‘*’ 0.05 ‘.’ 0.1 ‘ ’ 1.

### 3.2 Does Reproductive Success Vary With Carrion Substrate and/or Season?

We did not find any significant differences in the chance of brood failure across all our treatments, regardless of the time of collection in the field and the type of carrion the beetles bred upon (Table 2).

**TABLE 2:** Model summaries showing results of the models used to test for the effects of month trapped, carcass type used for breeding and their interaction on (a) brood success; (b) brood size; (c) average larval mass; (d) larval density and (e) carcass use efficiency of broods produced by *Nicrophorus vespilloides* beetles trapped in June and August 2017 and bred on chick carcasses and mice carcasses.

Fixed effects	Estimate	SE	*z* value	*p*r(> |*z*|)
*(a) Brood success (GLM)*				
Intercept	2.1001	0.4325	4.855	1.2e‐06***
Carcass bred on‐Mouse	0.5246	0.6337	0.828	0.408
Month trapped on‐June	1.1345	0.6886	1.648	0.099426
Carcass bred on‐Mouse × Month trapped on‐June	−0.7764	1.4818	−0.524	0.60032
*(b) Brood size (GLM)*				
Intercept	3.00964	0.04441	67.767	< 2e‐16***
Carcass bred on‐Mouse	−0.16545	0.07489	−2.209	0.027159*
Month trapped on‐June	0.28001	0.05937	4.716	2.4e‐06***
Carcass bred on‐Mouse × Month trapped on‐June	0.30736	0.08816	3.486	0.000489***
*(c) Average larval mass (LM)*				
Intercept	0.174569	0.006359	27.452	< 2e‐16***
Carcass bred on‐Mouse	0.009393	0.010180	0.923	0.3581
Month trapped on‐June	−0.002187	0.009086	−0.241	0.8103
Carcass bred on‐Mouse × Month trapped on‐June	−0.027644	0.012838	−2.153	0.0334*
*(d) Larval density (LM)*				
Intercept	0.61267	0.06057	10.12	< 2e‐16***
Carcass bred on‐Mouse	0.08731	0.07555	1.156	0.25
Month trapped on‐June	0.80361	0.07498	10.72	< 2e‐16***
Carcass bred on‐Mouse × Month trapped on‐June	0.29675	0.15457	1.920	0.0574
*(e) Carcass use efficiency (LM)*				
Intercept	10.718	0.830	12.91	< 2e‐16***
Carcass bred on‐Mouse	0.4102	1.0406	0.394	0.694
Month trapped on‐June	11.104	1.028	10.81	< 2e‐16***
Carcass bred on‐Mouse × Month trapped on‐June	2.659	2.149	1.237	0.218

Significance codes: 0 ‘***’ 0.001 ‘**’ 0.01 ‘*’ 0.05 ‘.’ 0.1 ‘ ’ 1.

**FIGURE A3:**
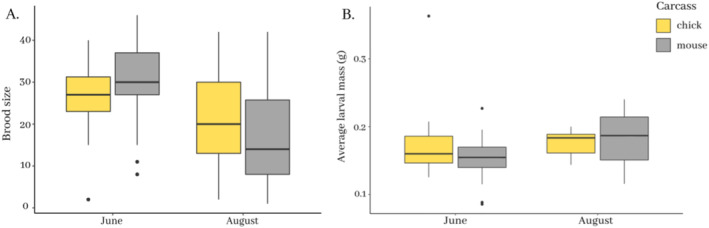
Fitness correlates of breeding *N. vespilloides* trapped on chick and mouse carrion in June and August 2017: (A) brood size and (B) average larval mass at dispersal of broods bred from adults trapped in June and August 2017 on chick carcasses (yellow bars) and mice carcasses (grey bars). The box bounds represent the inter‐quartile range (IQR), the whiskers represent 1.5 × IQR, the central horizontal line is the median, and the single points are outliers in the data.

However, we found that beetles trapped in June produced more surviving larvae than the August‐trapped beetles, regardless of the carrion they bred upon (Figure A3A, Table 2). In addition, we found that June‐caught beetles tended to produce even larger broods on mice than any other treatment (Table 2, Tukey post hoc comparison: *z* ratio = −3.051, *p*‐value = 0.0023). We also found that the June and August beetles had a different coefficient of variation in their brood size. Beetles bred in August had a greater coefficient of variation in brood size (CV = 0.605) compared to those bred in June (CV = 0.284; Test for equality of CV: test statistic = 26.38341, *p* < 0.0001).

We found a significant interaction between the month of trapping and the carrion type used for breeding on average larval mass at dispersal (Figure A3B, Table 2). When June‐trapped beetles were bred on mice, they produced smaller larvae than any other combination of trapping months and carrion type – probably because the larvae developed in a larger brood (Table 2, Tukey post hoc comparison: *t* ratio = 2.333, *p*‐value = 0.0214).

**FIGURE 2:**
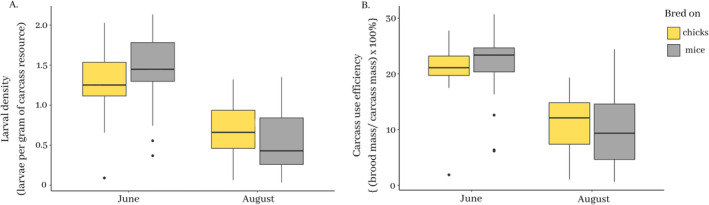
Reproductive success of *Nicrophorus vespilloides* trapped on chick and mouse carrion in June and August 2017 and measured in two different ways: (A) larval density and (B) carcass use efficiency. Adults were trapped in June and August 2017 in traps baited with either chick carcasses or mice carcasses. Adults were bred on the same carrion substrate they were trapped upon: Either dead chicks (yellow bars) or dead mice (grey bars). The box bounds represent the inter‐quartile range (IQR), the whiskers represent 1.5 × IQR, the central horizontal line is the median, and the single points are outliers in the data.

We used larval density and carcass use efficiency to further compare reproductive performance between June‐ and August‐trapped beetles as these two measures take into account the variation in carcass mass (Figure 2).

Broods bred from June‐trapped adults produced larvae at significantly higher density on the carcass compared to broods bred from August‐trapped adults (Figure 2A, Table 2). There was no significant effect of carcass type on larval density nor was there a significant interaction between the type of carcass the beetles bred on and the month in which the adults were trapped (Figure 2A, Table 2).

Beetles trapped in June utilised both chick and mouse carcasses significantly more efficiently than beetles trapped in August (Figure 2B, Table 2). There was no significant effect of carcass type on how efficiently beetles used the carcasses, nor any significant interaction between carcass type and sampling date. We found that beetles bred in August had a greater coefficient of variation in carcass use efficiency (CV = 0.320) compared to those bred in June (CV = 0.222; Test for equality of coefficients of variation: test statistic = 43.93225, *p*‐value < 0.0001).

By chance, beetles trapped in August 2017 were bred on significantly heavier carcasses in the laboratory (30.98 ± 2.1 S.D. (g)) than beetles trapped in June 2017 (21.20 ± 1.20 S.D. (g)), though comparing beetles trapped within each month, carcass mass was consistent between chick and mice treatments. The size range used in this experiment still corresponds with the size of carrion that *N. vespilloides* can use in nature (Müller, Eggert, and Dressel 1990; Otronen 1988).

### 3.3 Do Beetles That Are Attracted to Different Types of Carrion Also Differ Predictably and Seasonally in Their CHCs?

**FIGURE 3:**
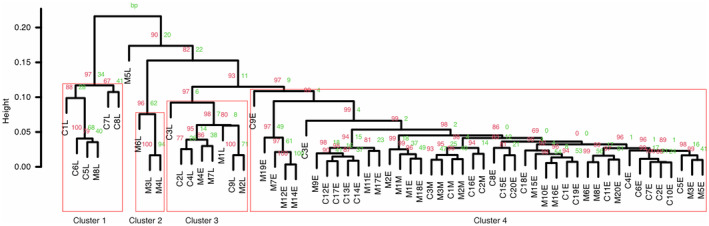
Hierarchical clustering of CHC profiles of *Nicrophorus vespilloides* beetles trapped on chick and mouse carrion at three time points during the 2017 field season. Cluster dendrogram with *p*‐values (%). Bootstrap probability values (BP) in green reflect the proportion of bootstrap samples where the same cluster is obtained. Approximately unbiased values (AU) in red are *p*‐values obtained by multiscale bootstrap resampling. Significant clusters with an AU *p*‐value greater than 0.95 (*p* < 0.05) are outlined in red. In the sample name, the first letter indicates the carrion type the individual was trapped on (C – chick, M – mouse). The following number is the sample ID, and the last letter indicates the trapping season (E – early, M – mid and L – late).

Our data revealed divergence in the CHC profiles of beetles trapped at different time points in the field season, which was greater than the divergence in CHC profiles between beetles trapped on different types of carrion (Figure 3, Table A2).

Of the four significant clusters in our data, the largest (Cluster 4) was composed of 44 beetles. All 6 mid‐season beetles were within this cluster, along with 38 early‐season beetles. Only 1 early season beetle lay outside of the first cluster, in Cluster 3, along with 7 late season beetles. Cluster 2 was the smallest cluster, composed of 3 late‐season beetles caught on mice. Cluster 1 contained 5 late‐season beetles caught on chicks and 1 late‐season trapped on a mouse.

**FIGURE A4:**
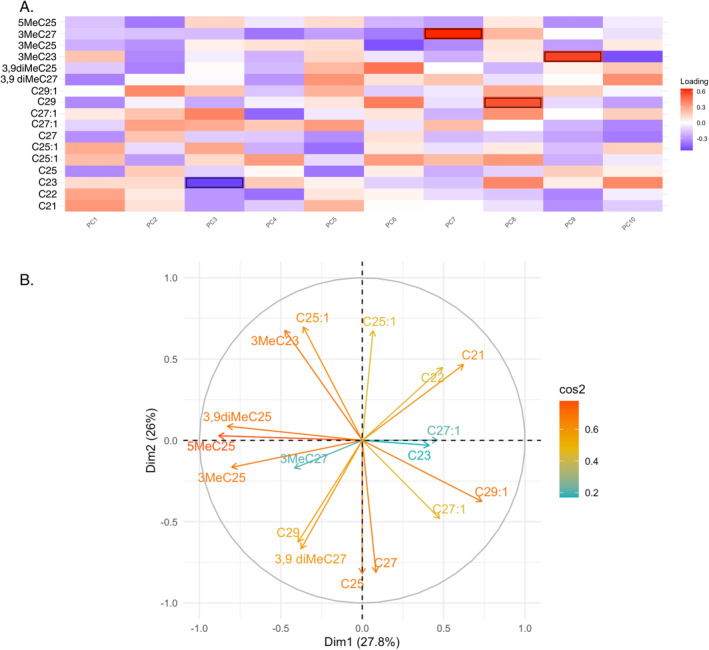
PCA of the CHCs of *N. vespilloides* beetles trapped on chick and mouse carrion at three time points during the 2017 field season. (A) Heatmap depicting the loadings of the first 10 Principal Components (PCs). Loadings represent the contribution of each original variable to the PC. The *x*‐axis indicates different PCs and the *y*‐axis represents different CHC compounds, which were the variables used in the PCA. The colour scale indicates the strength and direction (positive or negative) of each variable's contribution to the PCs. High absolute values suggest a strong contribution. Variables with positive loadings (in red) on a PC increase the score of that component and those with negative loadings (in blue) decrease the score of that PC. The midpoint of the colour gradient (in white) represents loadings near zero. Cells with loadings greater than ±0.5 have been outlined (in black). (B) Correlation between the first two PCs and each variable. Biplot depicting the squared cosine value (cos2) for each variable. Vectors with high cos2 values (in orange), that are closer to the grey correlation circle, are better represented by the component compared to vectors with low cos2 values (in blue). Consequently, the length of each vector indicates its contribution to the discrimination of samples based on their CHC profiles. The *x*‐axis is the first principal component and the *y*‐axis is the second principal component. Together the two axes explain 53.8% of the variation in the data.

The first 10 PCs of the PCA used to examine the CHC compounds found in our samples explained 96.3% of the variance in our data, with the first two PCs explaining 53.8% of this variance (Figure A4). The correlation plot between the first two PCs and each variable (Figure A4B) indicated that most of the CHCs had high squared cosine values and were well represented by the first two PCs. The hydrocarbons tricosane, nonacosane, 3‐methylheptacosane and 3‐methyltricosane had factor loadings higher than ±0.5 on one of the principal components (Figure A4A). Therefore, they contribute to the discrimination between significant clusters in beetle CHC profiles.

## 4 Discussion

We studied seasonal patterns of resource use in *N. vespilloides*, by manipulating resource availability on a local spatial scale in a Norfolk woodland using traps baited with mammalian and avian carrion. We compared the wild burying beetles collected in early and late summer and investigated whether the type of carrion resource that *N. vespilloides* beetles were trapped upon was associated with their reproductive success and differences in their cuticular hydrocarbons.

Beetles trapped in June were more likely to be found in the traps baited with mice whereas those trapped in August were equally likely to be found in mice‐baited and chick‐baited traps. If this trapping pattern reflects an adaptive preference for breeding on mice in June then beetles trapped in June should have greater reproductive success on mice over chicks. We did not find consistent support for this prediction. Beetles that were trapped in June and bred on mice produced more larvae than beetles in all other treatments (Figure A3A, Table 2). However, we also observed that beetles trapped in June had greater reproductive success in general and produced larvae at greater densities on both chick and mouse carcasses (Figure A3A, Figure 2A). Furthermore, June‐trapped beetles used both chick and mouse carcasses significantly more efficiently than beetles caught in August.

August‐trapped beetles were by chance bred on significantly larger carcasses, so it is important to consider the role that carcass size played in the results we observe and whether carcass size is a potential confounding effect. Previous work in other labs has shown that larger carcasses are generally associated with larger broods and heavier larvae (Bartlett and Ashworth 1988, Scott & Traniello 1990, Creighton 2005). Therefore, beetles from Thetford Forest behave in a similar way to other burying beetle populations. If their breeding performance was solely affected by carrion size, then August‐trapped beetles should have shown higher reproductive success than June‐trapped beetles. Yet we found the opposite pattern. We conclude, therefore, that our results are not caused by the August‐trapped beetles being bred on larger carrion. However, it is possible that slightly larger carrion is harder to process, and this could explain why June‐trapped beetles were able to breed more efficiently on the smaller carrion they were given to breed upon.

In another work (Issar 2021; Park, Issar, and Kilner 2024) using laboratory populations that were evolved on chick and mouse carrion, beetles bred on chicks also performed better than those bred on mouse carrion. Our results from laboratory populations show that chick carrion is a lower quality resource than mice carrion, possibly because the chick carrion we used was commercially produced and so might differ nutritionally from wild birds.

We found that June‐trapped beetles also produced larger broods on mice, comprising smaller larvae. However June‐trapped beetles did not produce more larvae per gram of carrion, or convert carrion more efficiently into larvae, than beetles trapped on chicks or caught in August. A likely explanation for these data is that the beetles trapped in June were not specialists in breeding on mice but rather were simply of higher quality than those trapped in August. August‐trapped beetles were more variable in their reproductive success, which suggests that the beetles breeding in August were in turn more variable in quality.

Just as with other insect species, there is likely to be seasonal variation in the age structure of natural *N. vespilloides* populations. In Europe, *N. vespilloides* adults emerge from overwintering in late spring. Presumably only higher quality individuals are able to survive the winter months and they might then breed twice in 1 year (Pukowski 1933; Scott 1998). Offspring from the first broods produced each year will have sufficient time to reach sexual maturity and produce one brood themselves before the annual breeding season comes to a close. Therefore by August, the breeding population is likely to comprise a combination of older adults and more recently eclosed individuals (Pukowski 1933; Urbański and Baraniak 2015): an instance of ‘generational smearing’ (Bjørnstad, Nelson, and Tobin 2016). This could account for the greater variation in the reproductive success that we observed in August‐trapped beetles.

Previous lab experiments on *N. vespilloides* have indicated that even when females switch strategies from reproductive restraint to terminal investment, older females have lower reproductive success due to senescence‐related constraints (Cotter, Ward, and Kilner 2011). Furthermore, work on natural populations of burying beetles has indicated a decline in brood mass in *N. orbicollis* populations later in the breeding season (Scott & Traniello 1990). An age‐structured population could partly explain why the quality of individuals in late summer was lower on average than earlier in the year. Whether this pattern exists in other *N. vespilloides* populations remains to be seen. Different populations could have different age structures in late summer, depending on local life history strategies and ecological conditions.

It is important to consider the limitations of our field and laboratory experiments. We measured differential resource use in the wild by actively manipulating resource availability on a local spatial scale in the woodland such that beetles were given a simultaneous choice between a dead mouse and a dead chick. It is not straightforward to compare our findings with previous work on resource use with other insects as most of these studies involve phytophagous insects such as fruit flies, moths and aphids where differential resource use can be quantified in a more natural way by simply measuring population density and occurrence on host plants (Feder et al. 1994; Groman and Pellmyr 2000; Via, Bouck, and Skillman 2000). Furthermore, our data are cross‐sectional snapshots at different moments in time through the breeding season. We were unable to track individuals to see how their behaviour varied across the season. It is possible that they follow a flexible carrion use strategy, due to the unpredictability and ephemerality of carrion as a resource.

Our analyses of beetle CHCs provide further evidence against the possibility of individual specialisation on particular carrion substrates and support for the alternative interpretation that there is instead seasonal variation in adult beetle quality. In *Nicrophorus* beetles, these cuticular compounds act as contact pheromones and they are an important means by which beetles recognise conspecifics and distinguish between the sexes (Steiger et al. 2007; Steiger, Peschke, and Müller 2008). In addition, beetles raising a brood use ‘breeding status’‐related CHC signatures to distinguish between their nestmate and an intruding conspecific (Müller et al. 2007; Steiger et al. 2007; Steiger, Peschke, and Müller 2008). The hydrocarbons tricosane, nonacosane, 3‐methylheptacosane and 3‐methyltricosane appear to contribute to the discrimination between significant clusters (Figure A4A) in our CHC data. Hydrocarbons with double bonds or methyl groups are more commonly associated with recognition cues than n‐alkanes such as tricosane (Howard and Blomquist 2005). 3‐methyl alkanes have been found to act as fertility signals in ants and social wasps (Holman, Lanfear, and D'Ettorre 2013; van Zweden et al. 2014), while the relative abundance of nonacosane is an indicator of age in female *Anopheles* mosquitoes (Brei et al. 2004). Therefore, these compounds could be possible markers of reproductive status or age in burying beetles.

We did not find a clear association between the CHC profiles of the beetles and the type of carrion they were trapped upon. It may be that individuals in the field are not sufficiently consistent in their use of carrion for there to be a carcass‐use‐related CHC signature. Alternatively, since diet‐related differences in CHCs are due to the incorporation of dietary hydrocarbons into cuticular lipids, it may be that the hydrocarbons derived from birds and mammals are insufficiently different to produce a diet‐based signature on the cuticle (Liang and Silverman 2000; Blomquist and Bagnères 2010; Otte, Hilker, and Geiselhardt 2014).

Nevertheless, we found that the CHC profiles of different beetles clustered according to the time of year when they were trapped (Figure 3). Furthermore, we found greater variation in the CHCs of late‐season beetles than early‐season beetles. Since the cuticular profiles of the beetle vary according to their reproductive state (Steiger et al. 2007; Scott 1998), this is consistent with our inference that there are seasonal differences in individual quality, age and breeding status within wild populations.

Many other insect species are also multivoltine, producing more than one generation in a year which can result in age‐structured populations (Wagner et al. 1984; Tauber and Tauber 1989; Gurney, Crowley, and Nisbet 1992; Molleman et al. 2006; Carey et al. 2008; Bjørnstad, Nelson, and Tobin 2016). Variation in age structure has been studied in depth for managing populations of insect pest species (Tauber 1989; Bonsall and Eber 2001; Cook, McMeniman, and O'Neill 2008; Rock, Wood, and Keeling 2015), but it is not yet known how it contributes to the dynamics of wild *N. vespilloides* populations.

Cyclic variation in rates of survival, reproductive success and developmental times in insect populations may be driven by fluctuations in the availability and quality of resources, interspecific competition, and the effects of abiotic environmental factors such as annual variation in temperature (Varley, Gradwell, and Hassell 1973; Plant and Wilson 1986; Haridas et al. 2016). Fitness in seasonal environments relies not only on the optimal timing of key life history events such as reproduction, hibernation or aestivation but also on the capacity to anticipate and prepare for seasonal changes before they occur (Bradshaw and Holzapfel 2007; Tsai et al. 2020). Recent work (Tsai et al. 2020) on locally adapted reproductive photoperiodism (i.e. differences in reproductive activity across seasons based on changes in day‐and‐night cycles) in the Asian burying beetle *N. nepalensis* has demonstrated that studying seasonal trends can help identify populations that could be especially vulnerable to future climate change. For example, Potticary et al. (2023) posit that seasonal variation in the competitive environments experienced by *Nicrophorus* spp. in woodlands of Clarke County, Georgia, USA has been influenced by climatic changes in the past two decades.

It is crucial to grasp how environmental fluctuations influence the dynamics of insect populations, particularly given that warming and rapid climate change are predicted to drastically affect species distribution and abundance globally (Meehl and Tebaldi 2004; Tylianakis et al. 2008; Paaijmans et al. 2013; Vasseur et al. 2014). In recent years, a growing number of studies have investigated the impacts of unnatural temperature shifts associated with global climate change on natural populations (Deutsch et al. 2008; Guo, Sun, and Kang 2011; Colinet et al. 2015; Stoks et al. 2017). Yet, several groups of insects are unrepresented in climate change research (Guo, Sun, and Kang 2011). Further work is needed to investigate how populations respond to natural variation in their biotic and abiotic environments to better predict long‐term species' responses to global change (Perez and Aron 2020). Our work has generated novel insight into how wild populations respond to fluctuations in resource availability and survival imposed by seasonal environments in one such understudied group of animals.

## Author Contributions

**Swastika Issar:** conceptualization (equal), data curation (lead), formal analysis (lead), funding acquisition (lead), investigation (lead), methodology (equal), validation (equal), visualization (lead), writing – original draft (lead), writing – review and editing (equal). **Chloé Leroy:** investigation (equal), methodology (supporting), validation (equal), visualization (equal), writing – review and editing (supporting). **Patrizia d'Ettorre:** investigation (equal), methodology (equal), resources (lead), validation (equal), visualization (equal), writing – review and editing (lead). **Rebecca M. Kilner:** conceptualization (equal), methodology (equal), project administration (lead), resources (lead), supervision (lead), writing – review and editing (lead).

## Conflicts of Interest

The authors declare no conflicts of interest.

### Open Research Badges

This article has earned an Open Data badge for making publicly available the digitally‐shareable data necessary to reproduce the reported results. The data is available on Dryad (DOI: https://doi.org/10.5061/dryad.8kprr4xvx).

## Data Availability

All our raw data and code are available to on Dryad (DOI: https://doi.org/10.5061/dryad.8kprr4xvx).

## Appendix A

**TABLE A1:** Identification of the 17 most regularly occurring peaks in the cuticular hydrocarbon profile of *Nicrophorus vespilloides*.

	Retention time (min)	Compound	Diagnostic EI ions (m/z)
Internal standard (IS)	7.47	C_18_ (Octadecane)	254
1	10.59	C_21_ (Heneicosane)	296
2	11.95	C_22_ (Docosane)	310
3	13.46	C_23_ (Tricosane)	324
4	14.57	3MeC_23_ (3‐methyltricosane)	57, 309, 281, 323
5	16.25	C_25:1_ (Pentacosene)	350
6	16.34	C_25:1_ (Pentacosene)	350
7	16.66	C_25_ (Pentacosane)	352
8	17.45	5MeC_25_ (5‐methylpentacosane)	85, 309, 281, 351
9	17.85	3MeC_25_ (3‐methylpentacosane)	57, 337, 309, 351
10	18.42	3,9diMeC_25_ (3,9dimethylpentacosane)	57, 155, 252, 351, 365
11	19.57	C_27:1_ (Heptacosene)	378
12	19.66	C_27:1_ (Heptacosene)	378
13	19.92	C_27_ (Heptacosane)	380
14	21.13	3MeC_27_ (3‐methylheptacosane)	57, 365, 337, 379
15	21.69	3,9 diMeC_27_ (3,9‐dimethylheptacosane)	57, 155, 281, 379, 393
16	22.81	C_29:1_ (Nonacosene)	406
17	23.17	C_29_ (Nonacosane)	408

*Note:* Diagnostic ions are provided.

**TABLE A2:** Activity season of field‐caught beetles within significant clusters differentiated by CHC profile.

Total beetles in the cluster	Number trapped during the early season	Number trapped mid‐season	Number trapped during late season
Cluster 1 (6)	0	0	6
Cluster 2 (3)	0	0	3
Cluster 3 (8)	1	0	7
Cluster 4 (44)	38	6	0
